# The cross-sectional and prospective associations between time-restricted eating and cardiometabolic health in community-dwelling adults: A systematic review and meta-analysis of observational studies

**DOI:** 10.1038/s41430-026-01755-w

**Published:** 2026-05-23

**Authors:** Viktoria Feit, Norbert Schmitz, Katharina Knaub, Lea-Marie Schick, Isabelle Mack, Mahdieh Shojaa

**Affiliations:** 1 Department of Population-Based Medicine, University Hospital Tübingen, Tübingen, Germany; 2 Department of Psychosomatic Medicine and Psychotherapy, University Hospital Tübingen, Tübingen, Germany

**Keywords:** Risk factors, Epidemiology, Cardiovascular diseases, Obesity, Endocrine system and metabolic diseases

## Abstract

**Background/Objectives:**

Well-established cardiometabolic disease (CMD) prevention strategies emphasize maintaining a balanced diet, regular physical activity, and a normal weight. The role of time-restricted eating (TRE; ≤12 h daily eating window) in improving cardiometabolic health remains elusive. This systematic review and meta-analysis of observational studies investigated the associations between TRE and cardiometabolic health in adults.

**Subjects/methods:**

We systematically searched PubMed, the Cochrane Library, Web of Science, and CINAHL for observational studies examining the association between TRE and cardiometabolic markers in adults for all articles published up to January 13, 2025. Risk of bias was assessed using the NIH Quality Assessment Tool. Qualitative and random-effects meta-analyses were performed if ≥3 studies reported the same outcome; otherwise, results were summarized narratively.

**Results:**

12 cross-sectional and 6 cohort studies were included. Meta-analysis of cross-sectional studies showed no significant association between TRE and abdominal obesity (OR = 0.96; 95% CI: 0.86–1.07; *I²* = 59%), metabolic syndrome (OR = 1.04; 95% CI: 0.90–1.19; *I²* = 32%), elevated blood pressure/hypertension (OR = 1.04; 95% CI: 0.971.12; *I²* = 0%), reduced high-density lipoprotein cholesterol (OR = 0.94; 95% CI: 0.83–1.06; *I²* = 78%), elevated triglycerides (OR = 1.06; 95% CI: 0.99–1.14; *I²* = 0%), and prediabetes/type 2 diabetes mellitus (OR = 1.04; 95% CI: 0.96–1.12; *I²* = 50%). Due to insufficient data, no conclusions could be drawn about prospective associations.

**Conclusions:**

To date, cross-sectional data have not consistently demonstrated associations between TRE and cardiometabolic health in community-dwelling adults. Until stronger real-world evidence emerges, the priority should remain on established CMD prevention strategies.

**Registry and registry number for systematic reviews or meta-analyses:**

PROSPERO registration number: CRD42023390410.

## Introduction

Cardiometabolic diseases (CMDs), including type 2 diabetes mellitus (T2DM), hypertension, and cardiovascular diseases (CVDs), are among the leading causes of death worldwide [1] and represent an economic and health burden [2]. The global number of prevalent cases of CMDs is expected to rise substantially, with T2DM cases projected to increase from 529 million (95% uncertainty interval: 500–564) in 2021 to 1.31 billion (95% uncertainty interval: 1.22–1.39) by 2050 [3]. Moreover, the number of deaths from CVDs is expected to increase worldwide by 73.4% by 2050, reaching 35.6 million [4].

Behavioral risk factors that contribute to the development of CMDs include smoking, physical inactivity, alcohol consumption, and a poor diet characterized by an inadequate intake of fruits and vegetables, as well as high consumption of salt, sugar, and fat [2, 5]. Risk assessment of CMDs typically includes established risk factors such as age, ethnicity, T2DM (hemoglobin A1c (HbA1c) >6.5%), abdominal obesity (waist circumference (WC) ≥102 cm in men and ≥88 cm in women), low high-density lipoprotein cholesterol (HDL-c; <40 mg/dL in men and <50 mg/dL in women), elevated triglycerides (TG; >150 mg/dL, nonfasting), elevated blood pressure (systolic blood pressure (SBP) of 120–139 mmHg or diastolic blood pressure (DBP) of 70–89 mmHg), and metabolic syndrome (MetS; three or more of the following risk factors: elevated TG, high blood pressure, increased WC, low HDL-c, and high glucose levels) [6–8]. Current guidelines on the primary prevention of CMDs emphasize lifestyle change, including a healthy diet, physical activity (PA), quitting smoking, and weight loss for individuals with overweight and obesity [6, 9, 10].

A potential strategy to improve metabolic health and reduce the risk of CMDs is intermittent fasting (IF). According to the International Consensus on Fasting Terminology, IF refers to repetitive fasting periods that can last up to 48 h [11]. IF includes different types of fasting regimes, including alternate-day fasting (fasting one day and eating unrestrictedly the next), periodic fasting (fasting one or two days a week and eating the rest of the week unrestrictedly), and time-restricted eating (TRE; consuming food only during a specific period) [11, 12].

Compared to other types of IF, TRE typically limits the daily eating duration (the number of hours between the first and last caloric consumption within 24 h [13]) to 12 h or less without additional caloric restriction [14, 15], accompanied by a nightly fasting duration (calculated as 24 h minus the eating duration [16, 17]) of more than 12 h [18]. While the alignment of circadian rhythms has been proposed as a mechanism through which TRE may improve cardiometabolic health [19], findings from clinical studies indicate that the benefits of TRE are linked to unintentional caloric restriction, weight loss [20], and improved glucose control [21]. A recently published umbrella review of systematic reviews and meta-analyses of randomized controlled trials (RCTs) found that IF, including TRE, has beneficial effects on several health outcomes in adults who are overweight or obese, including reductions in fasting insulin, low-density lipoprotein cholesterol (LDL-c), TG, total cholesterol, WC, and fat mass, while increasing HDL-c and fat-free mass [18]. Another systematic review and meta-analysis of clinical trials found similar benefits of TRE for adults with excessive weight and obesity-related metabolic diseases, including reduced fat mass, body weight, WC, blood glucose, insulin levels, and total cholesterol [22]. However, the effect of TRE on markers of cardiometabolic health has primarily been studied under controlled conditions and in highly selected populations such as adults with prediabetes, T2DM, overweight/obesity, and non-alcoholic fatty liver disease, often with small sample sizes, and short follow-up periods (the majority lasting 4–12 weeks, with only a few extending up to 1–2 years) [18]. Although RCTs are the gold standard for establishing causal effects, it remains elusive how these findings translate to real-world conditions and to the general population, where eating patterns, adherence, and health behaviors may differ.

Although TRE has predominantly been studied as an intervention in RCTs [23, 24], recent epidemiological studies have increasingly recognized TRE as a lifestyle factor, examining its associations with cardiometabolic health in populations [25, 26]. Despite the growing number of studies investigating cross-sectional [25, 26] and prospective associations between TRE and cardiometabolic outcomes [27], the evidence remains inconclusive. While several cross-sectional studies have reported a significant association between TRE and cardiometabolic health [25, 28], other studies have found no association [29] or a detrimental effect of a shorter daily eating window [26, 30]. For example, Currenti et al. (2021) showed that eating within a 10-h window was associated with lower odds of hypertension (odds ratio (OR) = 0.24; 95% CI: 0.13–0.45), dyslipidemia (OR = 0.26; 95% CI: 0.10–0.63), and with overweight/obesity (OR = 0.05; 95% CI: 0.01–0.07) [31], while Bernardes da Cunha et al. (2021) showed that adults with an eating duration of more than 12 h had a higher prevalence of abdominal obesity (incidence rate ratio = 1.15; 95% CI: 1.03–1.28). By contrast, another cross-sectional study found no significant association between fasting for over 12 h and MetS (OR = 1.30; 95% CI: 0.60–2.80) [32]. Furthermore, Wirth et al. (2021) reported that for each additional hour of nightly fasting duration, insulin levels significantly increased (β = 0.287; standard error = 0.057; *p* < 0.01), as did CRP levels (β = 0.020; standard error = 0.013; *p* = 0.02), while HDL-c levels significantly decreased (β = −0.103; standard error = 0.046; *p* = 0.03) [26].

In the context of well-established CMD prevention strategies, TRE has emerged as a potential additional lifestyle factor. RCTs consistently demonstrate that TRE improves weight and glycemic control, and lipid profiles when implemented as a protocol-driven intervention under controlled conditions. However, it is unclear whether these benefits extend to self-directed, uncontrolled settings. To address the gap, we conducted a systematic review and meta-analysis of observational studies examining the cross-sectional and prospective associations between TRE (defined as a daily eating window of 12 h or less and/or nightly fasting of more than 12 h) and markers of cardiometabolic health in community-dwelling adults.

## Methods

### Literature information sources, search strategy, and study selection

This systematic review was conducted following the Preferred Reporting Items for Systematic Reviews and Meta-Analysis (PRISMA) guidelines [33]. The review protocol was registered on the PROSPERO platform under the registration number CRD42023390410. The full strategy is available in the supplementary materials (Text S1, Search strategy, p.38 ff). The complete search strategy was developed with the support of an experienced librarian from the University Library of Tübingen, as was the translation of search terms from PubMed to other data platforms. A systematic search of the databases PubMed, Cochrane Library (CENTRAL) via Ovid, Web of Science, and CINAHL without language restrictions was performed to identify all observational studies investigating the association between TRE and cardiometabolic health. The search strategy included keywords such as “time-restricted eating”, “cardiometabolic risk factors”, and “observational studies” with Boolean operators (OR, AND) used to refine results.

The updated search was performed by two study authors (VF, KK) from inception to January 13, 2025, using a combination of Medical Subject Headings and free-text terms. All search results from the various databases were imported into EndNote reference software. The EndNote’s automatic duplicate detection function was used to identify and remove duplicates, followed by a manual check to ensure correctness. Following Bramer et al. (2017), the review of the retrieved references for inclusion was performed in two stages using EndNote [34]. First, three independent reviewers (VF, LMS, KK) screened all titles and abstracts. Second, the full-text articles of eligible studies were retrieved and independently screened by the same reviewers. In addition, the bibliographies of the studies that met the eligibility criteria were manually searched to identify other relevant references subjected to the same screening and selection process. Disagreements between reviewers during these two screening stages were resolved by discussion.

If a consensus could not be reached, a fourth reviewer was consulted (MS).

### Eligibility criteria

Eligibility criteria for this systematic review were defined according to the modified PEO (population, exposure, outcome) framework [35], with the addition of “S” for study design. Studies were included if they met the following criteria:

- **Participants:** This review included studies that focused on community-dwelling adults aged 18 years or older of all ethnicities and sexes and from all geographic regions. However, studies of pregnant, postpartum, and lactating women were excluded due to metabolic and hormonal changes and sleep disturbances [36], reduced sleep regularity, and dietary changes [37, 38]. To avoid bias, we excluded studies that included only special populations, such as participants with diabetes or other markers of cardiometabolic health.
- **Exposure:** Studies were eligible if they examined TRE as a categorical or continuous exposure. In the case of categorical exposures, TRE was defined as an eating duration of 12 h or less and a nightly fasting duration of more than 12 h. Studies that reported more than two categories of eating duration (e.g., based on quartiles) were included if at least one exposure group met the predefined inclusion criteria of an eating window of ≤12 h. In a few justified cases, studies with slight deviations from the threshold were included if they investigated TRE as the exposure and provided relevant comparisons against longer eating or shorter fasting durations. Examples include studies with a median eating duration of 12.7 h or a reported nightly fasting duration range of 10–12 h. In the case of continuous exposures (e.g., regression coefficients), studies were included independent of the exposure range to determine how variations in eating windows affected cardiometabolic markers. Studies investigating religious fasting, such as Ramadan fasting, were excluded because of changes in food intake and disrupted sleep-wake cycles [39].
- **Outcome**: We included studies reporting associations between TRE and (i) cardiometabolic markers or (ii) the risk of cardiometabolic disease. Studies that did not include these outcomes or reported unrelated outcomes were excluded.
- **Study type:** Only observational studies (e.g., cross-sectional studies and cohort studies) were included, whereas review articles, letters to the editor, commentaries, case reports, conference abstracts, and RCTs were excluded. RCTs were excluded because this review focuses on real-world behavior and aims to test if the evidence from controlled studies also applies to uncontrolled, real-world conditions.

### Data items, data selection, and organization

For each included study, two reviewers (VF, KK) extracted data on study characteristics (first author name, country, year of publication, sample size), study population characteristics (age, sex), exposure of interest (exposure type, assessment method), the cardiometabolic markers measured, statistical methods (type of analysis, adjustment for confounders), and results (unadjusted and adjusted effect estimates) [31]. Additionally, the follow-up duration (years) was summarized for cohort studies. If more than one statistical model with different adjustment levels was reported in the included studies, effect estimates were extracted from the fully adjusted model [32]. If data extraction parameters were missing, the corresponding authors were contacted.

As this systematic review aimed to assess both cross-sectional and prospective associations between TRE and cardiometabolic markers, results were grouped according to study design:

1. **Cross-sectional studies** included traditional cross-sectional studies and cross-sectional analyses derived from cohort studies.
2. **Cohort studies** included both prospective and retrospective designs.

In addition, studies were analyzed according to the markers of cardiometabolic health assessed. Abdominal obesity was defined using WC cut-offs that varied between studies and were based on regional and national standards. Definitions included cut-offs from North America (USA: WC >102 cm for men, >88 cm for women), the Middle East (Iran: WC ≥99.5 cm for men, ≥94.25 cm for women; Kuwait: WC ≥94 cm for men, ≥80 cm for women), East Asia (Japan: WC ≥90 cm for men, ≥80 cm for women; Korea: WC ≥90 cm for men, ≥85 cm for women). The cardiometabolic marker of prediabetes was grouped with the outcome of T2DM and defined as a fasting glucose level of ≥100 mg/dL, an HbA1c level of ≥5.6%, or a diagnosis of diabetes, or the use of antidiabetic medication or insulin. Elevated blood pressure was defined as having a SBP of 120–139 mmHg or a DBP of 70–89 mmHg [8]. The marker elevated blood pressure was combined with hypertension, defined as a SBP of ≥140 mmHg or DBP of ≥90 mmHg [8], or a previous diagnosis of hypertension or use of antihypertensive medication. MetS was defined as having three of the following criteria: elevated TG ( ≥150 mg/dL), elevated fasting glucose ( ≥100 mg/dL, with one study including HbA1c ≥5.6%), elevated blood pressure, reduced HDL cholesterol ( <40 mg/dL for men, <50 mg/dL for women), and abdominal obesity [6] (WC thresholds varied regionally). A TG level ≥150 mg/dL or being on medication was defined as having elevated TG. Reduced HDL-c was defined as an HDL-c level <40 mg/dL in men and <50 mg/dL in women, or for both sexes, an HDL-c level <50 mg/dL, or taking medication.

### Statistics

Qualitative and quantitative analyses were performed to synthesize the evidence from cross-sectional studies. When three or more studies were available for the same cardiometabolic outcome, a quantitative meta-analysis was conducted to assess the association between TRE and cardiometabolic health. Data analysis was performed using Review Manager (RevMan; version 5.4). A random-effects model was used, combining unadjusted ORs of studies comparing the TRE and no TRE groups. To make the studies comparable, the different exposure levels (e.g., eating/fasting windows, quartiles) were grouped by calculating the ORs of the combined subgroups. Therefore, the unadjusted effect estimates were used because some studies used different reference exposures and were not comparable. Another reason for using the raw data was that many studies reported estimates from various models adjusted for different confounders **(**Supplementary Appendix, Tables S1 and S2**)** [40]. Only studies that reported the number of cases and controls for each exposure category were included in the analysis. Heterogeneity among studies was assessed using the I^2^ metric, which was interpreted as not important (0–40%), moderate heterogeneity (30–60%), substantial heterogeneity (50–90%), and considerable heterogeneity (75–100%) [41].

In addition to the meta-analysis, a qualitative synthesis was conducted. This synthesis included studies that met the inclusion criteria but were not suitable for pooling for several reasons. First, some studies that were eligible for meta-analysis had incomplete statistical reporting. Second, there were methodological differences among the included studies in terms of populations, exposure definitions, and statistical analyses, among others. For instance, one study evaluated TRE as an eating duration of 8 h and 10 h (yes/no). However, the reference groups included eating durations within 9 and 12 h, making them non-comparable to studies with a clear TRE versus no-TRE contrast. Therefore, a qualitative analysis of the results was necessary for a complete interpretation of the findings. The inclusion criteria for the qualitative analysis were the same as for the quantitative analysis, requiring three or more studies for the same markers of cardiometabolic health. To maximize the comprehensiveness of the review and provide a clearer picture of the evidence, we conducted both analyses to ensure that all relevant data were included.

Narrative synthesis was performed for prospective and retrospective cohort studies, as well as for several markers of cardiometabolic health from cross-sectional studies, since meta-analysis was not feasible due to the presence of fewer than three studies for the same outcome.

### Risk of bias

Three reviewers (VF, LMS, KK) independently assessed the risk of bias for each study using the National Institute of Health (NIH) Quality Assessment Tool (available at: https://www.nhlbi.nih.gov/health-topics/study-quality-assessment-tools) for both cohort and cross-sectional studies [42], which has been used in another systematic review of observational studies [43]. The NIH quality assessment tool consists of the following 14 criteria: (1) research question, (2) study population, (3) participation rate, (4) groups recruited from the same population and with consistent eligibility criteria, (5) justification of sample size, (6) exposure assessed before outcome measurement, (7) sufficient time frame to see an effect, (10) repeated exposure assessment, (11) outcome measures, (12) blinding of outcome assessors, (13) follow-up rate, and (14) statistical analyses. Following the established guidelines for this assessment, studies were rated as good (11–14 out of 14 questions) with low risk of bias, fair (5–10 out of 14 questions) with moderate risk of bias, or poor (0–4 out of 14 questions) with high risk of bias. Disagreements about the risk of bias in individual studies were resolved through discussion among the reviewers. If a consensus was not reached, a fourth reviewer (MS) was consulted.

## Results

### Study selection

**Fig. 1:**
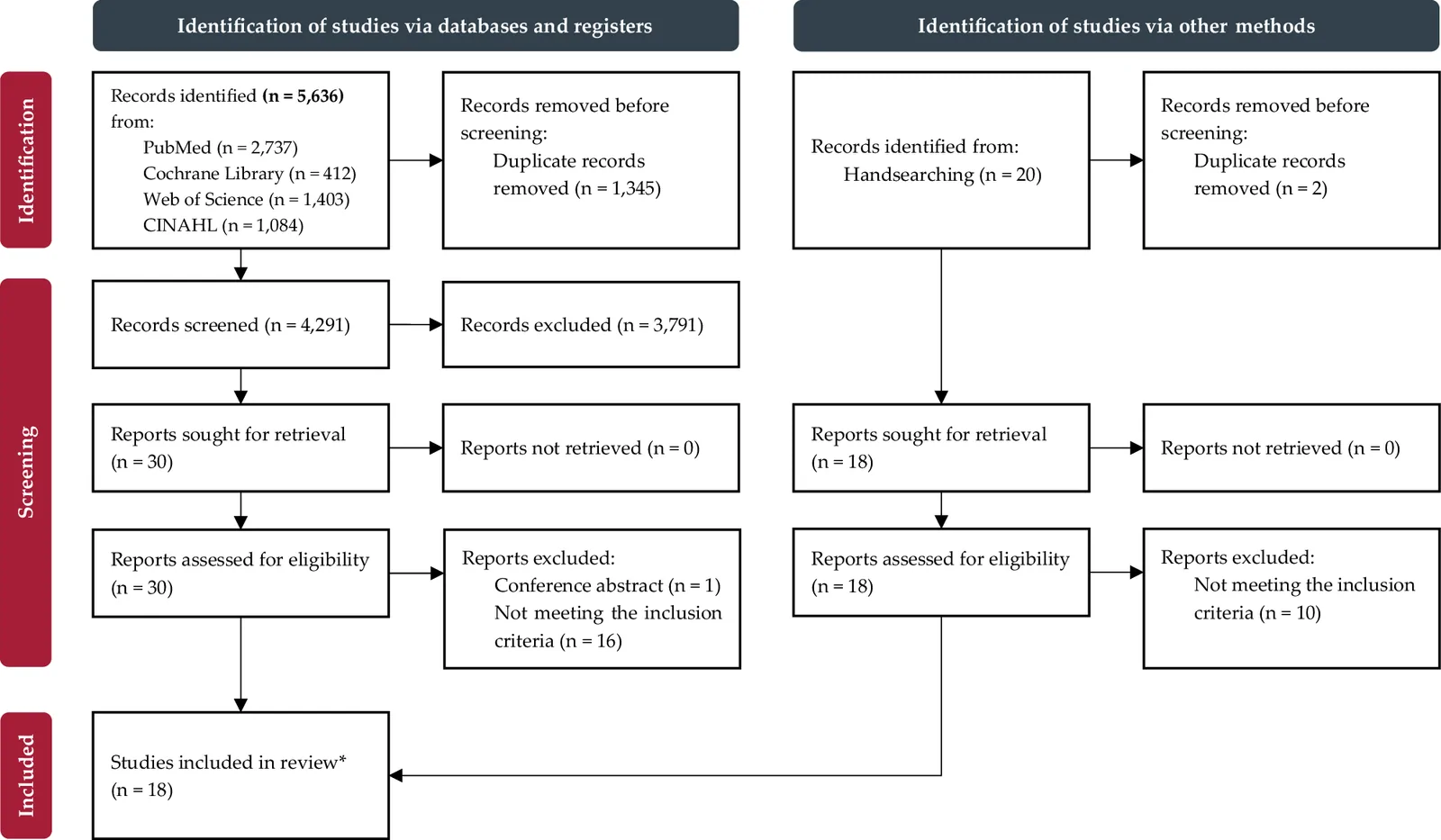
PRISMA flow diagram for search strategy. Combinations: 1st combination: 2 publications combined into one entry [44, 45]; 2nd combination: 2 publications combined into one entry [30, 46]. 3rd combination: 2 publications combined into one record [27, 47]. Created according to Page et al. (2021) [30].

The flow diagram of the literature search process is shown in Fig. 1. Out of 5,636 records, 18 studies were included in the systematic review. This number considers the application of three combining actions, in which pairs of studies with similar study teams, study populations, and participant numbers were combined into single records. As a result, the six studies were merged into three. The first combination action included both studies by Marinac and colleagues published in 2015, based on data from the US National Health and Nutrition Examination Survey (2009–2010). In both publications, the full eligible sample consisted of 2212 adult women [44, 45]. The first publication, released in May 2015, examined the association between nightly fasting duration and 2-h glucose concentration and HbA1c [44]. The second publication, published in August 2015, examined the relationship between nightly fasting duration and C-reactive protein, as well as the Homeostatic Model Assessment for Insulin Resistance [45]. The second combination action included two publications by Makarem et al. from 2020 and 2021, based on data from an American Heart Association Go Red for Women Strategically Focused Research Network ancillary study [30, 46]. The first study published in 2020, with a sample size of 116 at baseline, investigated cross-sectional and prospective associations of habitual nightly fasting duration with cardiometabolic risk factors and cardiovascular health [30]. The second study, published in the following year, included 115 study participants at baseline and examined the relationship between the nightly fasting duration and markers of cardiometabolic health [46]. The third combination action combined two studies by Palomar-Cros and colleagues, both published in 2023 and based on the NutriNet-Santé study, a prospective cohort study from France [27, 47]. One study investigated the association between nightly fasting duration and the risk of CVD with a total of 103,389 study participants and a median follow-up of 7.2 years [47]. Similarly, the second publication analyzed the relationship between nightly fasting duration and the incidence of T2DM in 103,312 study participants with a median follow-up of 7.3 years [27].

### Summary of study characteristics

In total, 12 studies were cross-sectional [16, 17, 25, 26, 29, 32, 44, 45, 48–52] and six cohort studies (five prospective and one retrospective) [27, 28, 30, 46, 47, 53–55]. Among the cohort studies, two studies analyzed the cohort data only cross-sectionally [54, 55], and one study [30, 46] combined cross-sectional and prospective analyses, thereby being listed in both study types. The search did not identify any case-control studies.

The missing data included a lack of information on the mean age of the groups assessed, the number of exposed and unexposed individuals, and the number of individuals with and without the outcome. Additionally, there were missing p-values for the associations between the different outcomes. Of the 14 study authors contacted, five (35.71%) provided additional information.

In the following, the characteristics of the studies and the main results are presented by study type.

### Cross-sectional studies

Table 1 shows the characteristics and main results of the 15 included cross-sectional studies and cross-sectional analyses of cohort studies. The majority of these studies examined populations from the US (*n* = 8, 53.33%) [26, 30, 44–46, 48, 49, 51, 52, 54], followed by Korea (*n* = 2, 13.33%) [17, 50], Japan [29], Iran [16], Kuwait [32], Spain [55], and Italy [25] (each *n* = 1, 6.67%), with the number of participants ranging from 115 in one study [30, 46] to 38,302 in another study [51]. In addition, five studies utilized data from the National Health and Nutrition Examination Survey [48] and two used data from the Korea National Health and Nutrition Examination Survey [17, 50]. The mean age ranged from 33 years [30, 46] to a weighted mean of 70.75 years [55], with one study not reporting the mean age [51]. The majority of studies included both sexes, and two studies included women only [30, 44–46].

**Table 1:** Characteristics of cross-sectional studies and cross-sectional analyses of cohort studies evaluating the association between TRE and markers of cardiometabolic health.

author (year)	country	sample size (*n*)	subgroup (*n*)	age, mean (SD)	sex, % female	assessment of TRE (*n*)	TRE duration	marker(s) and main results
Ali et al. [48]	USA	7619	total population (7,619)	49.8 (17.9)	51.5	24-h dietary recall (n = 2)	eating duration: ≤10 h vs. >12–13 h	glucose ↔ HOMA-IR ↔
Bernardes da Cunha et al. [49]	USA	7379	adults (4,879) elderly (2,500)	38.35 (12.4) 69.60 (6.8)	51.3 49.4	24-h dietary recall (n = 2)	eating duration: >12 h vs. ≤12 h	obesity ↔ abdominal obesity ↓* (adults) MetS ↔ elevated blood pressure ↔ reduced HDL-c ↔ elevated TG ↔ elevated glucose ↔
Currenti et al. [25]	Italy	1936	eating duration: ≤8 h (108) eating duration: >8 h (1,828) eating duration: ≤10 h (276) eating duration: >10 h (1,660)	54.7 (15.7) 48.1 (17.6) 49.2 (17.5) 43.8 (17.1)	56.5 58.6 55.1 59.0	FFQs (n = 2); meal timing assessment (past 6 months)	eating duration: ≤10 h vs. >10 h	overweight/obesity ↓^†^ hypertension ↓^†^ T2DM ↔ dyslipidemia ↓^†^
Estrada-deLeón et al. [55]	Spain	1047	nightly fasting duration: <10 h (488) nightly fasting duration: 10 to < 12 h (341) nightly fasting duration: ≥12 h (218)	70.6 (3.8) 70.5 (3.7) 71.5 (4.2)	47.0 46.0 57.0	computerized diet history; 24-h dietary recall (n = 7)	nightly fasting duration: ≥12 h vs. <10 h	cholesterol ↔ HDL-c ↓^**^ (difference) LDL-c ↔ TG ↔ glucose ↔ insulin ↔ HbA1c ↔ hs-CRP ↔
Ha and Song [50]	Korea	14,279	men (5,854) women (8,425)	41.1 (0.2) 41.7 (0.2)	0 100	24-h dietary recall (n = 1)	nightly fasting duration: 10–12 h vs. ≥16 h	obesity ↔ MetS ↔ abdominal obesity ↓^*^ elevated blood pressure ↔ reduced HDL-c ↔ elevated TG ↓^*^ elevated glucose ↔
Kwak et al. [17]	Korea	22,685	nightly fasting duration (men): 8.1 ± 0.1 h (2,680) nightly fasting duration (men): 11.1 ± 0.01 h (1,954) nightly fasting duration (men): 12.5 ± 0.01 h (2,728) nightly fasting duration (men): 15.6 ± 0.1 h (2,321) nightly fasting duration (women): 9.4 ± 0.04 h (3,524) nightly fasting duration (women): 11.9 ± 0.01 h (3,073) nightly fasting duration (women): 13.4 ± 0.01 h (3,627) nightly fasting duration (women): 16.1 ± 0.04 h (2,778)	45.4 (0.4) 49.6 (0.4) 49.9 (0.4) 41.3 (0.4) 48.5 (0.3) 50.6 (0.3) 50.1 (0.4) 42.0 (0.4)	0 0 0 0 100 100 100 100	24-h dietary recall (n = 1)	men, nightly fasting duration: ≥12.0 h vs. <12 h women, nightly fasting duration: ≥12.17 h vs. <12.7 h	T2DM ↓^*^ (for men)
Marinac et al. [45] and Marinac et al. [44]	USA	2212	nightly fasting duration: <11.5 h (770) nightly fasting duration: 11.5–13.49 h (746) nightly fasting duration: ≥13.5 h (770)	48.4 (1.0) 46.6 (0.9) 45.5 (0.7)	100 100 100	24-h dietary recall (n = 1)	nightly fasting duration: continuous (in h increase/percentage change per unit increase)	HbA1c ↔ elevated HbA1c ↓^†^ (each 3-h increase in nightly fasting duration) elevated 2-h glucose levels ↔ 2-h glucose ↓^*^ (percent change per unit increase in nightly fasting duration) CRP ↔ HOMA-IR ↔
O’Connor et al. [51]	USA	38,302	total population (38,302)	NR	50.2	24-h time use diary (n = 1)	eating duration: continuous (per one h increase)	normal weight ↑^***^ (AP per one-hour increase of eating duration) overweight ↓^***^ (AP per one-hour increase of eating duration) obesity ↓^*** (AP^ per one-hour increase of eating duration)
Ueda et al. [29]	Japan	1054	nightly fasting duration: ≥12 h (247) nightly fasting duration: =11 h (321) nightly fasting duration: =10 h (275) nightly fasting duration: ≤9 h (211)	46.3 (10.4) 47.0 (9.5) 45.1 (8.0) 45.5 (7.7)	17.8 12.8 10.2 7.6	self-reported dinner and breakfast timing (usual time)	nightly fasting duration: ≥12 h vs. =11 h, =10 h, ≤9 h	MetS ↔ high WC ↔ elevated TG ↔ reduced HDL-c↔ elevated blood pressure ↔ elevated blood glucose ↔
Wirth et al. [26]	USA	15,939	nightly fasting duration: <10.75 h (7,164) nightly fasting duration: 10.75–12.16 h (8,231) nightly fasting duration: 12.17–13.75 h (7,333) nightly fasting duration: ≥13.75 h (7,226)	48.3 (0.30) 47.7 (0.28) 47.8 (0.39) 43.9 (0.38)	44.0 52.0 57.0 55.0	24-h dietary recall ( n ≥ 1)	nightly fasting duration: >12.16 h vs. ≤12.16 h	elevated CRP ↔ elevated HbA1c ↔ elevated insulin ↔ elevated glucose ↔ elevated HDL-c ↔ elevated LDL-c ↔ elevated cholesterol ↔
Zeng et al. [52]	USA	3813	eating duration: ≥10 h (2,566) eating duration: 8–10 h (511) eating duration: ≤8 h (736)	51.4 (17.4) 48.7 (19.6) 42.7 (19.1)	51.9 57.6 50.8	24-h dietary recall (n = 2)	eating duration: 8–10 h, ≤10 h vs. ≥10 h	NAFLD ↓ (eating duration ≤ 8 h vs. ≥ 10 h)
Zeraattalab-Motlagh et al. [16]	Iran	850	total population (850)	44.7 (10.6)	68.7	24-h dietary recall	nightly fasting duration: ≥11.25 h vs. <11.25 h	abdominal obesity ↔ increased blood pressure ↔ decreased HDL-c ↔ elevated TG ↓^*^ increased fasting glucose ↔ MetS ↓^*^
Makarem et al. [46] and Makarem et al. (2020)^2^ [30]	USA	115 116	total population (115–116)	33 (12)	100	24-h dietary recall	nightly fasting duration: continuous (per 30 min/1 h increase)	BMI ↔ WC ↔ LDL-c ↔ HDL-c ↔ SBP ↔ DBP ↔ fasting glucose ↔ HbA1c ↔
Alkhulaifi et al.[32]	Kuwait	757	total population (757)	37.8 (12.3)	55.0	24-h dietary recall	nightly fasting duration: 10–12 h, >14 h vs. <8 h	MetS ↔ abdominal obesity ↔ elevated blood pressure ↔ low HDL-c ↔ elevated TG ↓^†^ (nightly fasting duration 8–10h vs. < 8h) elevated fasting glucose ↔
Xiao et al. [54]	USA	994	nightly fasting duration: median 9.5 h (201) nightly fasting duration: median 10.6 h (197) nightly fasting duration: median 11.3 h (196) nightly fasting duration: median 12.1 h (201) nightly fasting duration: median 13.4 h (199)	61.8 (5.4) 63.0 (6.1) 61.8 (6.5) 62.8 (6.2) 64.5 (5.3)	32.6 49.4 48.5 57.4 52.1	24-h dietary recall (n = 6)	median nightly fasting duration: Q4(12.1 h) vs. Q1(9.5 h)	overweight/obesity ↔

*AP* adjusted prevalence, *BMI* body mass index, *CRP* C-reactive protein, *DBP* diastolic blood pressure, *FFQ* food frequency questionnaire, *HbA1c* hemoglobin A1c, *HDL-c* high-density lipoprotein cholesterol, *HOMA-IR* homeostasis model assessment of insulin resistance, *LDL-c* low-density lipoprotein cholesterol, *MetS* metabolic syndrome, *NAFLD* non-alcoholic fatty liver disease, *SBP* systolic blood pressure, *T2DM* type 2 diabetes mellitus, *TG* triglycerides, *TRE* time-restricted eating, *WC* waist circumference. Statistical significance for all outcomes, except where indicated, is determined by p-values, with significance indicated by symbols (*p* < 0.05, *p < 0.01, ***p < 0.0001). † For this specific outcome, *p*-values were not available, and statistical significance was determined based on 95% confidence intervals (CIs). ^1^Marinac et al. [44] and Marinac et al. [45] were assessed as a single study. ^2^Makraem et al. [30] and Makarem et al. [46] were appraised as one study. ↔, indicates no significant association; ↓ indicates a significant negative association (*p* < 0.05); ↑ indicates a significant positive association (*p* < 0.05).

TRE was evaluated as nightly fasting duration in 10 studies [16, 17, 26, 29, 30, 32, 44–46, 50, 55] and eating duration in five studies [25, 48, 49, 51, 52]. TRE was assessed by 24-hour dietary recall in 12 studies [16, 17, 26, 30, 32, 44–46, 48–50, 52, 54, 55], by food frequency questionnaire and meal timing assessment in one study [25], by 24-hour time use diary in one study [51], and by self-reported dinner and breakfast timing in one study [29]. Nine studies assessed the association between TRE and markers of cardiometabolic health by group comparison (TRE vs. no TRE or different levels of eating duration/nightly fasting duration compared to the reference) [16, 25, 29, 32, 49, 50, 52, 54, 55], three studies examined the relationship by looking at the dose-response relationship between TRE and cardiometabolic markers [30, 44–46, 51], and three studies combined a group comparison with a dose-response assessment [17, 26, 48]. In addition, three studies examined the effect of the timing of TRE on cardiometabolic health [48, 52, 54]. Furthermore, two studies investigated the joint association between nightly fasting duration and lifestyle factors with markers of metabolic health [50, 52]. While Ha and Song (2019) explored the joint association between nightly fasting duration and sleep duration with MetS and obesity [50], Zeng et al. (2023) investigated the association between nightly fasting duration and nonalcoholic fatty liver disease according to PA [52]. The most commonly studied cardiometabolic markers were elevated blood pressure/hypertension [16, 25, 29, 32, 49, 50], and elevated glucose [16, 26, 29, 32, 49, 50], each assessed in six studies, followed by MetS [16, 29, 32, 49, 50] and reduced HDL [16, 29, 30, 32, 46], assessed in five studies each.

### Cohort studies

Characteristics of prospective and retrospective cohort studies evaluating the association between TRE and markers of cardiometabolic health are shown in Table 2. Two studies used cohorts from the US [30, 46, 53], one study a cohort from the US and Canada [28], and one study from France [27, 47]. The mean age at baseline ranged from 33 years [30, 46] to a weighted mean of 58 years [28], and the number of participants ranged from 115 [30, 46] to 103,389 [27, 47]. The follow-up duration of these studies ranged from one year [30, 46] to a median follow-up time of 10 years [53]. Three studies included men and women in their analysis [27, 28, 47, 53], while one study included only women [30, 46]. Three studies assessed TRE using 24-hour dietary recall [27, 30, 46, 47], and one study used self-reported meal timing [28]. The prospective association between TRE and cardiometabolic markers was assessed using group comparison over time in two studies [27, 47, 53], as a change in outcome per year in one study [28], and as a prospective dose-response relationship in one study [30, 46]. In addition, one study examined the relationship between TRE and the incidence of T2DM, considering the timing of breakfast (before or after 8 am) [27]. Three studies evaluated metabolic markers (body mass index (BMI), WC, fasting glucose, HbA1c, and T2DM) [27, 28, 30, 46], two studies evaluated cardiovascular markers (LDL-c, HDL-c, DBP, SBP, overall CVD, cerebrovascular disease, and coronary heart disease) [30, 46, 47], and one study evaluated various causes of mortality (all-cause mortality, CVD mortality, heart disease-specific mortality, and stroke-specific mortality) [53] and their association with TRE.

**Table 2:** Characteristics of prospective and retrospective cohort studies evaluating the association between TRE and cardiometabolic health markers.

author (year)	country	follow-up (years)	sample size (*n*)	subgroup (*n*)	baseline age, mean (SD)	sex, % female	assessment of TRE (*n*)	TRE duration	marker(s) and main results
Kahleova et al. [28]	USA, Canada	7.4 (mean)	50,660	NA	58 (13)	61.9	self-reported meal timing	nightly fasting duration: 18–24 h vs. 12–17 h	BMI ↓^***^ (nightly fasting duration 18–24 h, change per year)
Makarem et al. [30] and Makarem et al. (2021)¹ [46] [	USA	1.0 (fixed)	116 115	NA	33 (12)	100	24-h dietary recall	nightly fasting duration: continuous (per 30 min/1 h increase)	BMI ↔ WC ↔ LDL-c ↔ HDL-c ↔ SBP ↔ DBP ↔ fasting glucose ↔ HbA1c ↔
Palomar-Cros et al. [27] and Palomar-Cros et al. [47]	France	7.3 (median) 7.2 (median)	103,312 103,389	NA	42.7 (14.6) 42.6 (14.5)	78.9 79.0	24-h dietary recall ( n ≥ 3)	nightly fasting duration: 12–13 h, >13 h vs. ≤12 h nightly fasting duration: continuous (per 1 h increase)	T2DM ↔ overall CVD ↔ cerebrovascular diseases ↓^*^(nightly fasting duration 12–13 h vs. ≤12 h) coronary heart disease ↔
Cheng et al. [53]	USA	10.0 (median)	30,646	nightly fasting duration: ≤10 h (7896) nightly fasting duration: 10–11 h (5116) nightly fasting duration: 11–12 h (5480) nightly fasting duration: 12–14 h (6505) nightly fasting duration: ≥14 h (5467)	46.8 (0.32) 47.9 (0.32) 47.3 (0.32) 45.9 (0.39) 42.8 (0.42)	45.0 49.7 54.3 56.2 55.1	24-h dietary recall (n = 2)	nightly fasting duration: 12–14 h, ≥14 h vs. 10–11 h	all-cause mortality ↑^†^ (nightly fasting duration 12–14 h, ≥14 h vs. 10–11 h) CVD mortality ↑^†^ (nightly fasting duration ≥14 h vs. 10–11 h) heart disease-specific mortality ↑^†^ (nightly fasting duration ≥14 h vs. 10–11 h) stroke-specific mortality ↑^†^ (nightly fasting duration ≥14 h vs. 10–11 h)

*BMI* body mass index, *CVD* cardiovascular disease, *DBP* diastolic blood pressure, *HbA1c* hemoglobin A1C, *HDL-c* high-density lipoprotein cholesterol, *LDL-c* low-density lipoprotein cholesterol, *NA* not applicable, *SBP* systolic blood pressure, *SD* standard deviation, *T2DM* type 2 diabetes mellitus, *TRE* time-restricted eating, *WC* waist circumference. Statistical significance for all outcomes, except where indicated, was determined using *p*-values, with significance indicated by symbols (*p* < 0.05, *p < 0.01, ***p < 0.0001). † For this specific outcome, *p*-values were not available, and statistical significance was determined based on 95% confidence intervals (CIs). An association was considered statistically significant if the CI did not include 1.00. ^1^Makraem et al. [30] and Makarem et al. [46] were assessed as a single study. ^2^Palomar-Cros et al. [47] and Palomar-Cros et al. [27] were considered as one study. ↔, indicates no significant association, ↓, indicates a significant negative association (*p* < 0.05); ↑ indicates a significant positive association (*p* < 0.05).

### Summary of study outcomes

#### Cross-sectional associations between TRE and cardiometabolic health

**Fig. 2:**
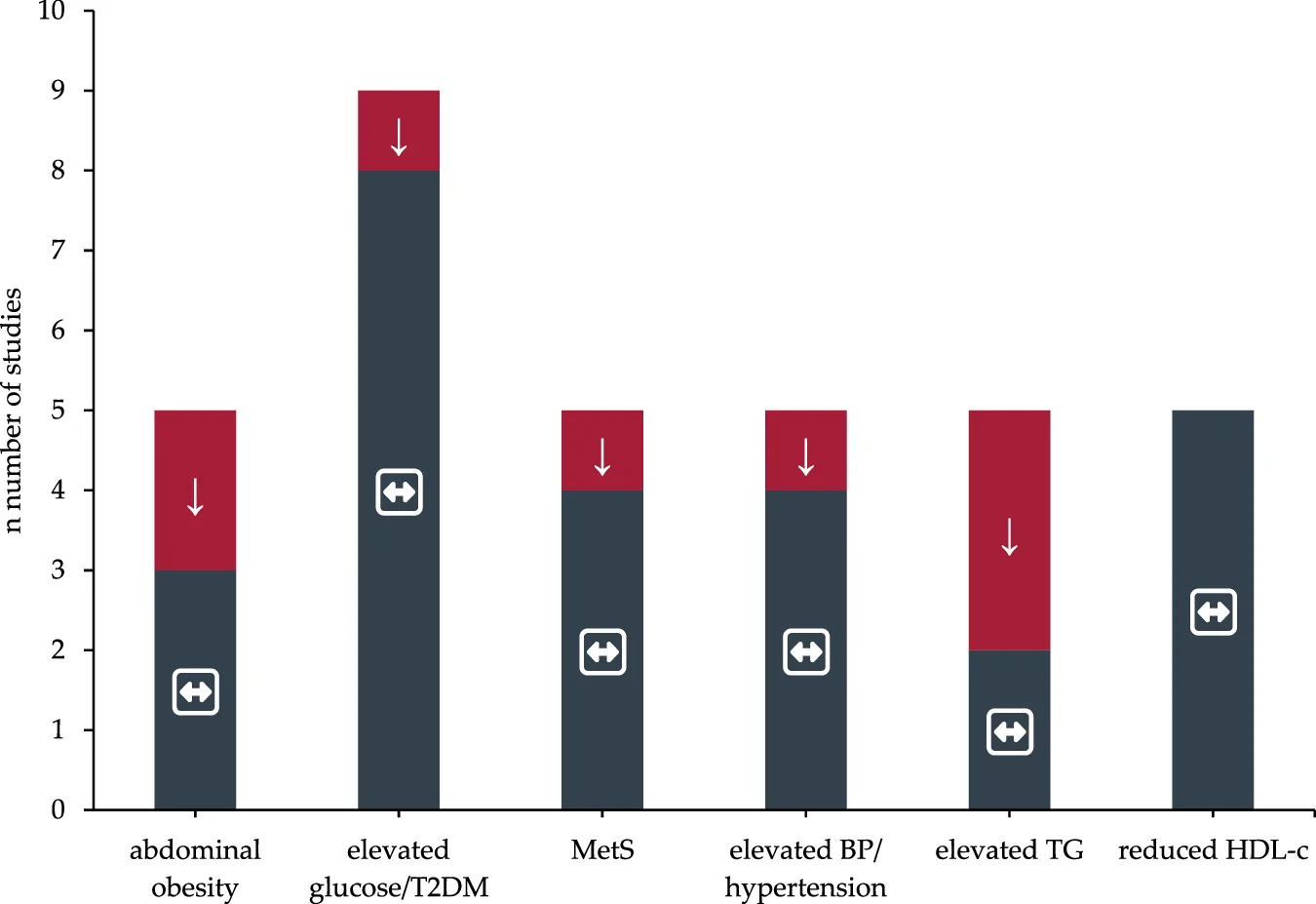
Qualitative association between nightly fasting duration and markers of cardiometabolic health (abdominal obesity, elevated glucose/T2DM, MetS, elevated blood pressure/hypertension, elevated TG, reduced HDL-c) across heterogeneous studies. Results from studies that assessed TRE as daily eating durations were converted to nightly fasting duration to provide a clearer understanding of the main markers of cardiometabolic health in the cross-sectional studies. In addition, results from studies that used different comparison groups and reference categories were summarized. For abdominal obesity, significant negative associations were found by Ha and Song (2019) [50] (nightly fasting duration: 10–12 h vs. ≥16 h) and Bernardes da Cunha et al. (2023) [49] (adults: nightly fasting duration <12 h vs. ≥12 h), while three studies found no significant association [16, 29, 32]. For prediabetes/T2DM, eight studies found no significant association with nightly fasting duration [16, 17, 25, 26, 29, 32, 49, 50], and Kwak et al. (2023) [17] showed a significant negative association (men nightly fasting duration ≥12 vs. <12 h). One study found a significant negative association between nightly fasting duration and MetS (NFD <11.25 h vs. NFD ≥11.25 h) [16], while four studies showed no association [29, 32, 49, 50]. Regarding elevated blood pressure/hypertension, Currenti et al. (2021) ^†^[25] showed a significant negative association (adults: nightly fasting duration ≥14 h vs. <14 h), and five studies showed no significant association [16, 29, 32, 49, 50]. Regarding elevated TG, three studies found a significant negative association with nightly fasting duration (Alkhulaifi et al. (2024)†: nightly fasting duration 8–10 h vs. <8 h [32]; Zeraattalab-Motlagh et al. (2023): nightly fasting duration ≥11.25 h vs. <11.25 h [16]; Ha and Song (2019), men: nightly fasting duration 10–12 h vs. ≥16 h [50]) and two studies found no significant association [29, 49]. Five studies examined the relationship between nightly fasting duration and reduced HDL-c and found no significant association [16, 26, 29, 49, 50]. ↔, indicates no significant association, ↓, indicates a significant negative association (*p* <0.05). ^†^ indicates significance based on 95% CIs. Definition of cardiometabolic health markers: Abdominal obesity, definitions included cut-offs from North America (USA: WC >102 cm for men, >88 cm for women [49]), the Middle East (Iran: WC ≥99.5 cm for men, ≥94.25 cm for women [16]; Kuwait: WC ≥94 cm for men, ≥80 cm for women [32]), East Asia (Japan: WC ≥90 cm for men, ≥80 cm for women [29]; Korea: WC ≥90 cm for men, ≥85 cm for women [50]); Prediabetes/T2DM, defined as a fasting glucose level of ≥100 mg/dL, or an HbA1c level of ≥5.6%, or a diagnosis of diabetes or use of antidiabetic medication or insulin; MetS, defined as the three of the following criteria being fulfilled: elevated TG (≥150 mg/dL), elevated fasting glucose (≥100 mg/dL, with one study including HbA1c ≥5.6%), elevated blood pressure, reduced HDL-c (<40 mg/dL for men, <50 mg/dL for women), and abdominal obesity (WC thresholds varied regionally); Elevated blood pressure was defined as a SBP of 120–139 mmHg or a DBP of 70–89 mmHg. Hypertension was defined as a SBP of ≥140 mmHg or a DBP of ≥90 mmHg, or a previous diagnosis of hypertension, or use of antihypertensive medication; elevated TG, defined as a TG level of ≥150 mg/dL or being on medication; Reduced HDL-c, defined as an HDL-c level <40 mg/dL in men and <50 mg/dL in women, or for both sexes, an HDL-c level <50 mg/dL or on medication. *DBP* diastolic blood pressure, *HbA1*c hemoglobin A1c, *HDL-c* high-density lipoprotein cholesterol, *MetS* metabolic syndrome, *T2DM* type 2 diabetes mellitus, *TRE* time-restricted eating, *SBP* systolic blood pressure, *TG* triglycerides, *WC* waist circumference.

Figure 2 provides an overview of the heterogeneous associations between nightly fasting duration and cardiometabolic markers (different group comparisons) at a qualitative level based on markers assessed in at least three studies. The results illustrate the distribution of negative and nonsignificant associations. The most common outcome examined in the included cross-sectional studies was prediabetes/T2DM. Among these studies, seven studies found no significant association with nightly fasting duration [16, 25, 26, 29, 32, 49, 50], and Kwak et al. (2023) [17] showed a negative association between nightly fasting duration and T2DM (in men: nightly fasting duration ≥12.0 h vs. <12.0 h). For the markers of cardiometabolic health, MetS, and elevated blood pressure/hypertension, the majority of the studies found no significant association [16, 29, 32, 49, 50]. However, Zeraattalab-Motlagh et al. (2023) showed a negative association between nightly fasting duration and MetS (nightly fasting duration <11.25 h vs. ≥11.25 h) [16], and Currenti et al. (2021) found a negative association between nightly fasting duration and elevated blood pressure/hypertension (nightly fasting duration ≥14 h vs. <14 h) [25]. Regarding abdominal obesity, Zeraattalab-Motlagh et al. (2023) [16], Ueda et al. (2021) [29], and Alkhulaifi et al. (2024) [32] showed no significant association with nightly fasting duration, whereas two studies found a significant negative association [49, 50]. While Ha and Song [50] showed significantly lower odds of abdominal obesity in men (OR = 0.70, 95% CI: 0.53–0.92) and women (OR = 0.74, 95% CI: 0.56–0.98) when comparing a nightly fasting duration of 10-12 h with a nightly fasting duration of ≥16 h, Bernardes da Cunha et al. (2023) [49] showed significantly lower odds in adults (OR = 0.87, 95% CI: 0.78–0.97) but not in the elderly comparing an nightly fasting duration <12 h with an nightly fasting duration of ≥12 h. For the relationship between nightly fasting duration and elevated TG, three studies found a significant negative association, with comparisons including nightly fasting duration 8–10 h vs. <8 h (OR = 0.47; 95% CI: 0.24–0.91) [32], nightly fasting duration ≥11.25 h vs. <11.25 h (OR = 0.73; 95% CI: 0.55–0.98) [16], and nightly fasting duration 10–12 h vs. ≥16 h (men, OR = 0.69; 95% CI: 0.53–0.91; women, OR = 0.93; 95% CI: 0.68–1.28) [50], and three studies found no significant association [29, 32, 49]. No significant associations were found between reduced HDL-c and nightly fasting duration [16, 26, 29, 49, 50]. The studies were heterogeneous in several terms, including varying exposure duration (e.g., nightly fasting duration ≥11.25 h vs. <11.25 h in one study [16] and adults: nightly fasting duration ≥14 h vs. <14 h in another study) and population differences (e.g., different countries, including Korea, the USA, Kuwait, Iran, Japan, and Italy). Furthermore, although all the included cross-sectional studies controlled for confounders, these differed from study to study, making it challenging to make comparisons. The most common confounders were age (*n* = 15, 100%), education (*n* = 11, 73.33%), smoking (*n* = 11, 73.33%), PA (*n* = 10, 66.67%), and energy intake (*n* = 10, 66.67%). 60% (*n* = 9) of the studies controlled for sex and 46.67% (*n* = 7) controlled for sleep duration (Supplementary Appendix, Table S1).

**Fig. 3:**
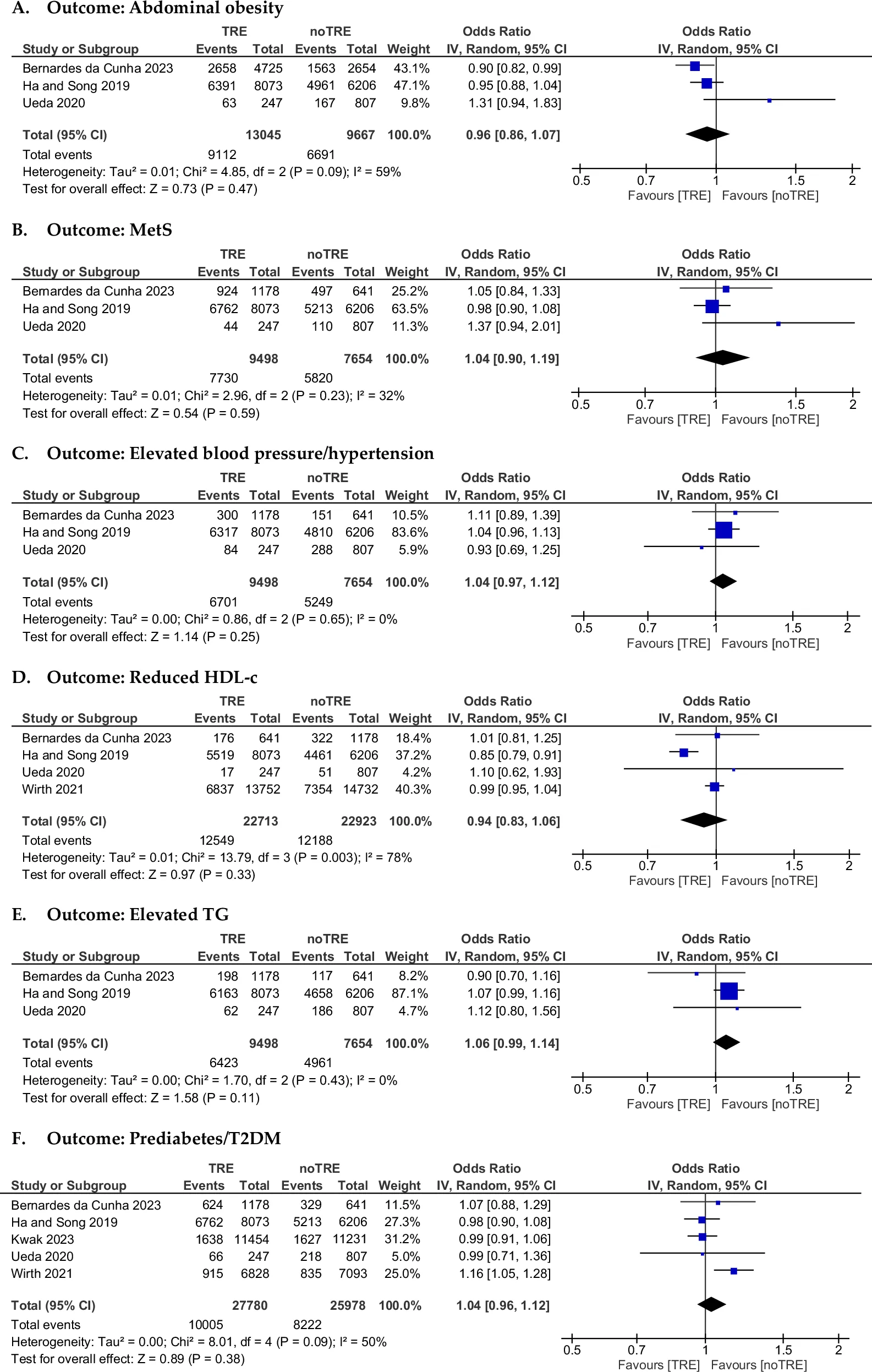
Quantitative analysis of cross-sectional studies evaluating the association between TRE (nightly fasting duration ≥12 h vs. nightly fasting duration <12 h) and markers of cardiometabolic health. Presented are unadjusted odds ratios (ORs) with 95% confidence intervals that have been harmonized across studies. Reported outcomes were converted to ORs when necessary for consistency. For studies with stratified results (e.g., by age or sex) or with more than two exposure groups, the participant numbers were aggregated into exposed/non-exposed and event/no-event categories. Additionally, exposure groups were combined where appropriate to enable a consistent comparison of TRE versus no TRE. Meta-analysis for **A** abdominal obesity [29, 49, 50], defined as WC >102 cm for men, >88 cm for women for USA, as WC ≥90 cm for men, ≥85 cm for women for Korea, and as a WC ≥90 cm for men, ≥80 cm for women for Japan; **B** MetS [29, 49, 50], defined as the presence of three of the following criteria: elevated TG (≥150 mg/dL), elevated fasting glucose (≥100 mg/dL, with one study including HbA1c ≥ 5.6%), elevated blood pressure, reduced HDL-c (<40 mg/dL in men, <50 mg/dL in women), and abdominal obesity (WC thresholds varied regionally); **C** elevated blood pressure/hypertension [29, 49, 50], where elevated blood pressure was defined as a SBP between 120 and 139 mmHg or a DBP between 70 and 89 mmHg, and hypertension was defined as a SBP of at least 140 mmHg or a DBP of at least 90 mmHg, or a previous diagnosis of hypertension or use of antihypertensive medication; **D** reduced HDL-c [26, 29, 49, 50], defined as an HDL-c level <40 mg/dL in men and <50 mg/dL in women, HDL-c level <50 mg/dL in both sexes or use of medication; **E** elevated TG, defined as a TG level ≥150 mg/dL or being on medication [29, 49, 50]; and **F** prediabetes/T2DM [17, 26, 29, 49, 50] was defined as a fasting glucose level of ≥100 mg/dL, or an HbA1c level of ≥5.6%, or a diagnosis of diabetes or use of antidiabetic medication or insulin. Bernardes da Cunha et al. (2023) [49], adults and elderly: nightly fasting duration >12h vs. ≤12h; Ha and Song (2019), [50], men and women: nightly fasting duration ≥12 h vs. <12 h; Kwak et al. (2023) [17], men and women: nightly fasting duration ≥12 h vs. <12 h for men and ≥12 h vs. <12 h for women; Ueda et al. (2021) [29], nightly fasting duration ≥12 h vs. =11 h, =10 h, ≤9h; Wirth et al. (2021) [26], nightly fasting duration >12.16 h vs. ≤12.16 h. Zeraattalab-Motlagh et al. (2023) [16] and Alkhulaifi et al. (2024) [32] were not included in the analysis because they did not provide supplementary data. Currenti et al. (2021) [25] were not included in the analysis due to different eating window subgroups. *DBP* diastolic blood pressure, *HbA1c* hemoglobin A1c, *HDL-c* high-density lipoprotein cholesterol, *MetS* metabolic syndrome, *T2DM* type 2 diabetes mellitus, *TRE* time-restricted eating, *SBP* systolic blood pressure, *TG* triglycerides, *WC* waist circumference.

In addition to the qualitative analysis, a quantitative analysis was performed for six cardiometabolic markers, each assessed in three or more studies (see Fig. 3). The number of studies included for each of the markers was variable: three studies each for abdominal obesity, MetS, elevated blood pressure/hypertension, and elevated TG [29, 49, 50], four studies for reduced HDL-c [26, 29, 49, 50], and five studies for prediabetes/T2DM [17, 26, 29, 49, 50]. Random-effects models were used, and the estimated summary effects were presented as ORs. No significant associations with nightly fasting duration were found for any of the six cardiometabolic health markers analyzed, as shown in Fig. 3. Due to insufficient data across studies, subgroup analyses could not be performed. Therefore, it was not possible to examine potential differences in the association between nightly fasting duration and markers of cardiometabolic health due to factors such as sex and age.

### Prospective associations between TRE and cardiometabolic health

#### Metabolic health (BMI, weight, and T2DM)

Looking at metabolic markers, Kahleova et al. (2017) showed that after a median follow-up time of 7.4 years, the BMI of study participants with a nightly fasting duration of more than 18 h decreased significantly compared to those with a nightly fasting duration of 12 to 17 h (p-trend <0.001) [28]. However, another study found no significant prospective association between nightly fasting duration and BMI in women after one year of follow-up [30, 46]. No significant associations were found between TRE and other metabolic markers [27, 30, 46]. However, in a prospective study (*n* = 103,312) with a median follow-up time of 7.3 years, Palomar-Cros et al. (2023) showed that participants with a nightly fasting duration longer than 13 h and who ate breakfast before 8 am had a significantly lower incidence of T2DM (hazard ratio (HR) = 0.47; 95% CI: 0.27–0.82) [27].

#### Cardiovascular risk markers (elevated blood pressure/hypertension, lipids, CVD)

Regarding cardiovascular markers, Palomar-Cros et al. (2023) found that the risk of cerebrovascular disease was inversely associated with nightly fasting duration (HR = 0.93; 95% CI: 0.87–0.99; *p*-value = 0.02) [47]. In contrast, no significant association was found between TRE and blood lipid levels, elevated blood pressure/hypertension, coronary heart disease, or total CVD [30, 46, 47].

#### Mortality outcomes

Furthermore, a limited number of studies evaluated mortality outcomes. Cheng et al. (2024) found a U-shaped association between nightly fasting duration and cardiovascular and all-cause mortality. Study participants with nightly fasting duration shorter than 10 h or longer than 14 h had a significantly higher risk of CVD (HR= 1.30; 95% CI: 1.08-1.55 for ≤10 h; HR= 1.37; 95% CI: 1.12–1.67 for ≥14 h), all-cause (HR= 1.23; 95% CI: 1.08–1.39 for ≤10 h; HR= 1.36; 95% CI: 1.19–1.54 for ≥14 h), stroke-specific (HR= 1.89; 95% CI: 1.11–3.23 for ≤10 h; HR= 1.84; 95% CI: 1.04–3.24 for ≥14 h) and heart-specific mortality (HR= 1.41; 95% CI: 1.11–1.79 for ≤10 h; HR= 1.48; 95% CI: 1.12–1.97 for ≥14 h) [53].

All of the included cohort studies controlled for confounders, with age being the most commonly controlled for (*n* = 4, 100%), followed by education, sex, race/ethnicity, PA, and energy intake, all of which were controlled for in three out of four studies. Only half of the studies controlled for smoking, alcohol consumption, and sleep duration (Supplementary Appendix, Table S2). No meta-analysis was performed for cohort studies because no more than two studies examined the same outcome.

#### Risk of bias

The risk of bias was assessed using the NIH quality assessment tool, and the results are shown in Table 3. In addition, we visualized the results of the risk-of-bias assessment, which can be found in the Supplementary Appendix**(**Figs. 1, 2**)**. The overall quality of all 12 included cross-sectional studies was rated as fair. A shared limitation of all included cross-sectional studies was the missing or inadequate justification of sample size (Q5), followed by the lack of blinding of outcome assessors (Q12) and follow-up rate (Q13), which did not apply to all studies. Moreover, most cross-sectional studies lacked a sufficient timeframe to observe an effect (Q7; ten studies did not fulfill this criterion, while two did) and did not include a repeated exposure measure (Q10; nine studies did not meet this criterion, while one did).

**Table 3:** NIH Quality Assessment Tool.

author (year)	Q1	Q2	Q3	Q4	Q5	Q6	Q7	Q8	Q9	Q10	Q11	Q12	Q13	Q14	Quality rating
***cross-sectional studies***															
Ali et al. (2023) [48]	Yes	Yes	NR	Yes	No	Yes	Yes	Yes	Yes	Yes	Yes	No	NA	Yes	Fair
Bernardes da Cunha et al. (2023) [49]	Yes	Yes	No	Yes	No	No	No	No	Yes	No	Yes	NR	NA	Yes	Fair
Currenti et al. (2021) [25]	Yes	Yes	Yes	Yes	No	Yes	Yes	Yes	No	No	Yes	NR	NA	Yes	Fair
Ha and Song (2019) [50]	Yes	Yes	Yes	Yes	No	Yes	No	Yes	Yes	No	Yes	NR	NA	Yes	Fair
Kwak et al. (2023) [17]	Yes	Yes	Yes	Yes	NA	No	No	Yes	Yes	No	Yes	NR	NA	Yes	Fair
Marinac et al. (2015) [44] and Marinac et al. (2015)^1^ [45]	Yes	Yes	NA	Yes	NR	NA	No	Yes	Yes	No	Yes	No	NA	Yes	Fair
O’Connor et al. (2022) [51]	Yes	Yes	Yes	Yes	No	No	No	Yes	Yes	No	Yes	NR	NA	Yes	Fair
Ueda et al. (2021) [29]	Yes	Yes	Yes	Yes	No	Yes	No	Yes	No	No	Yes	NR	NA	Yes	Fair
Wirth et al. (2021) [26]	Yes	Yes	NA	Yes	No	Yes	No	Yes	Yes	No	Yes	NR	NA	Yes	Fair
Zeng et al. (2023) [52]	Yes	Yes	No	Yes	No	Yes	No	Yes	Yes	No	Yes	NR	NA	Yes	Fair
Zeraattalab-Motlagh et al. (2023) [16]	Yes	Yes	NR	Yes	No	No	No	No	Yes	No	Yes	NR	NA	Yes	Fair
Alkhulaifi et al. (2024) [32]	Yes	Yes	NA	Yes	No	No	No	Yes	Yes	No	Yes	NR	NA	Yes	Fair
***cohort studies***															
Estrada-deLeón et al. (2022) [55]	Yes	Yes	Yes	Yes	No	Yes	Yes	Yes	Yes	No	Yes	No	NR	Yes	Fair
Xiao et al. (2021) [54]	Yes	Yes	NA	Yes	No	No	No	Yes	Yes	Yes	Yes	NR	NR	Yes	Fair
Kahleova et al. (2017) [28]	Yes	Yes	NR	Yes	No	Yes	Yes	Yes	No	No	Yes	NR	NR	Yes	Fair
Makarem et al. (2021) [30] and Makarem et al. (2020)^2^ [46]	Yes	Yes	Yes	Yes	No	Yes	Yes	Yes	Yes	Yes	Yes	NR	Yes	Yes	Good
Palomar-Cros et al. (2023) [27] and Palomar-Cros et al. (2023)^3^ [47]	Yes	Yes	Yes	Yes	No	Yes	Yes	Yes	Yes	Yes	Yes	NR	Yes	Yes	Good
Cheng et al. (2024) [53]	Yes	Yes	NA	Yes	No	Yes	Yes	Yes	Yes	No	Yes	NR	NR	Yes	Fair

^1^Marinac et al. [45] and Marinac et al. [44] were assessed as one study. ^2^Makarem et al. [30] and Makarem et al. [46] were evaluated as one study. ^3^Palomar-Cros et al. [27] and Palomar-Cros et al. [47] were assessed as one study. *NA* not applicable, *NR* not reported. Quality assessment criteria: Q1, Research question; Q2 and Q3, Study population; Q4; Groups recruited from the same population and uniform eligibility criteria; Q5, Sample size justification; Q6, Exposure assessed prior to outcome measurement; Q7, Sufficient.

Of the six cohort studies, four were rated as fair [28, 53–55], and two as good [27, 30, 46, 47]. None of the studies reported sample size justification (Q5), and the majority of the studies did not report blinding of outcome assessors (Q12). In addition, four studies [28, 53–55] did not report the follow-up rate (Q13), whereas two did [27, 30, 46, 47]. Further sources of bias included the lack of repeated exposure assessment (Q10) in three cohort studies, the absence of an exposure assessment before the outcome measure (Q6), insufficient time to observe an effect (Q7), and unclear exposure measurement and assessment (Q9) in one study each.

## Discussion

The major modifiable risk factors for CMDs include smoking, hypertension, obesity, and T2DM, and current guidelines emphasize a balanced diet, regular exercise, and weight management [6, 9]. Within this context, TRE has emerged as a potential lifestyle approach to support cardiometabolic health. Preclinical trials have shown that time-restricted feeding improves cardiometabolic health by reducing body weight, protecting against hepatic steatosis, improving glucose tolerance, lowering blood pressure, and atherogenic lipids [56]. Similarly, RCTs have consistently demonstrated that TRE, when implemented as a fasting protocol, improves markers of cardiometabolic health, including total cholesterol, HbA1c, insulin concentrations, body weight, and inflammation [19, 57, 58]. These findings strongly support the effectiveness of TRE under controlled conditions, both clinically and mechanistically.

In contrast, our meta-analysis of cross-sectional studies in community-dwelling adults found inconsistent associations between TRE and markers of cardiometabolic health. Restricting the daily eating window to 12 h or less was not consistently associated with cardiometabolic health. Additionally, we observed a qualitative pattern of lower prevalence of elevated TG with longer nightly fasting in three of the five studies. However, this pattern was not confirmed in the meta-analysis, probably due to heterogeneity and limited poolable data (i.e., incomplete statistical reporting and missing group counts). Therefore, while the qualitative analysis highlights a potential association, the findings remain limited and require further evidence for stronger conclusions.

The discrepancy between TRE as a closely monitored clinical intervention and TRE as a naturally occurring, self-directed lifestyle behavior in observational studies likely stems from differences in (i) variations in population characteristics (selected populations vs. community-dwelling adults), (ii) differences in exposure control (protocolized, closely monitored windows vs. self-directed behaviors), (iii) varying adherence (protocolized vs. self-directed), and (iv) residual or unmeasured confounding factors (e.g., sleep quality, stress, and shift work). Additionally, most observational studies relied on one or two 24-h dietary recalls (see Table 1), which may not accurately capture fasting patterns (e.g., intentional or not) and can result in misclassification of exposure, which could weaken the associations found in RCTs.

Unlike under controlled conditions, where adherence to TRE is high, timing and food intake are monitored, and confounding variables are minimized [59, 60], observational studies reflect the complexity of real-world behaviors. Extensive evidence indicates that cardiometabolic health is influenced by multiple lifestyle factors, including sleep patterns, PA, stress levels, dietary composition [61–64], and their interactions [65]. Adhering to several healthy lifestyle behaviors simultaneously has been associated with a 60% lower risk of stroke and a 66% lower risk of CVD, with a dose-response effect [66]. Although most of the included studies controlled for some lifestyle factors, there may still be unmeasured confounding (e.g., sleep quality and chronic stress).

Nevertheless, a recent Cochrane Review from 2026, analyzing 22 RCTs (including cluster RCTs), suggests that intermittent fasting may result in little to no difference in weight loss, quality of life, or adverse events compared to standard dietary advice. Compared to no intervention, the effects are also small and often uncertain [67]. Overall, intermittent fasting did not show clear advantages, and its use should therefore be guided by individual feasibility and long-term sustainability.

Moderate and high heterogeneity were observed across studies in the meta-analysis of abdominal obesity, MetS, prediabetes/T2DM, and reduced HDL-c. This variability likely reflects variations in the definition of TRE (e.g., cutoffs for eating/fasting durations), population demographics (e.g., age distribution, sex, or ethnicity), assessment methods, and cultural dietary norms. For example, the three studies included in the analyses of abdominal obesity and MetS were based on populations from the USA [49], Korea [50], and Japan [29], which vary in demographics, lifestyles, and cultural dietary norms. Furthermore, the cutoff values used to define TRE varied across the studies [25, 49, 50], and none of the studies included in the meta-analysis considered the timing of TRE (e.g., early vs. late), despite clinical evidence suggesting that earlier eating windows may be more beneficial for metabolic outcomes [68].

Emerging evidence from observational studies suggests that the duration [69] and timing of the eating window may further modify associations between TRE and cardiometabolic health [48, 54]. For example, Cheng et al. (2024) found a U-shaped relationship between TRE and cardiometabolic health, with both short ( <10 h) and long ( >14 h) eating windows associated with an increased risk of CVD and all-cause mortality [53]. Moreover, one large cohort study (*n* = 103,312 adults) in our review found that nightly fasting was inversely associated with the incidence of T2DM only when the first meal was consumed early in the day (at 8 am or before) [27]. Similarly, findings from RCTs have shown that earlier TRE (consuming dinner or the last meal before 4 pm) and shorter eating windows (4–6 h) result in more favorable effects on several markers of cardiometabolic health in adults with overweight/obesity or obesity-related metabolic diseases [22, 68]. These findings suggest that future research on TRE should consider not only eating/fasting duration, but also chrononutritional factors, such as timing and alignment with circadian rhythms.

Our review also highlights a significant gap in cohort studies investigating the association between TRE and the risk of cardiometabolic diseases, such as the incidence of T2DM and CVDs. Without longitudinal data, it remains elusive how TRE affects cardiometabolic health in the long term. Future studies should address this gap through study designs that better capture the timing and intentionality of eating/fasting windows, evaluate adherence over time, and consider the interaction of TRE with other chrononutritional and lifestyle factors in real-world settings.

### Strengths and limitations

This systematic review and meta-analysis has several strengths and limitations. A major strength of this review is that it focused on real-world observational data in community-dwelling adults, providing insight into the association between TRE, as a lifestyle factor, and cardiometabolic health in uncontrolled settings. This complements the evidence base from preclinical and clinical trials. Additionally, this review was conducted following the PRISMA guidelines and applied a comprehensive search strategy. To increase the homogeneity, only observational studies that defined TRE as a daily meal window of ≤12 h and/or a nightly fasting duration of >12 h were included.

Although there were clear eligibility criteria, the included studies were heterogeneous in terms of age, sex, exposure assessment, cardiometabolic markers included, statistical analysis, confounders controlled, and different levels or groups of exposures compared, which posed a challenge for systematic comparison. However, this was partially addressed by presenting the data additionally qualitatively. In observational studies, TRE assessment typically relies on self-reported data, which may be influenced by reporting bias and changes in dietary intake over time, as well as variations in eating behavior between weekdays and weekends. Moreover, most of the included studies investigated TRE using one or two 24-h dietary recalls, which do not necessarily reflect habitual eating or fasting patterns and can therefore result in misclassification.

Although cross-sectional studies provide useful snapshots of associations, their design presents challenges due to their inability to establish causality, susceptibility to reverse causation, difficulty tracking adherence over time, and limited ability to control for unmeasured confounding factors. Although all of the included studies reported adjusted effect estimates, the meta-analysis was conducted with unadjusted effect estimates, which are susceptible to confounding factors. This approach was selected to ensure consistency across studies because adjusted estimates were reported inconsistently, were based on different sets of confounders, and were often not directly comparable due to stratification or varying reference groups.

We conducted a meta-analysis on six markers of cardiometabolic health to ensure a comprehensive synthesis of the available evidence. However, we observed moderate to high heterogeneity across studies, which may reflect differences in study populations, measurement methods, or definitions. However, subgroup meta-regression to investigate potential effect modifiers (e.g., gender, age, and the timing or duration of TRE) was not possible due to the small number of studies and the insufficient reporting of the necessary stratified results.

This review was designed to evaluate TRE as a lifestyle exposure in free-living populations rather than as a protocolized intervention. Accordingly, our findings should be interpreted along with clinical trials and mechanistic evidence, highlighting the translation from intervention efficacy to real-world effectiveness. These limitations highlight opportunities for future research to incorporate longitudinal designs and more objective measures to strengthen the evidence base on TRE and cardiometabolic markers.

### Implications for practice and policy

The widespread popularity of TRE suggests that individuals are looking for simple, flexible, and sustainable dietary approaches to improve their cardiometabolic health. While TRE has shown promise for improving metabolic and cardiovascular health in controlled settings and specific populations, current observational studies in community-dwelling adults have not shown a uniform pattern of beneficial associations. This contrast underscores that while TRE may be biologically effective, its translation into population-level benefits depends on adherence, precision of exposure measurement, and interactions with other lifestyle factors. Future research should incorporate wearable technology to record real-time adherence and eating patterns and examine the effects of TRE on populations with varying meal timing habits. Until stronger real-world evidence is available, prioritizing well-established CMDs prevention strategies – such as avoiding smoking and alcohol, maintaining a healthy balanced diet, engaging in regular exercise, reducing sedentary behavior, managing stress, and maintaining normal body weight, as recommended by the American College of Cardiology/American Heart Association [6] and the European Society of Cardiology [9] – remains the most evidence-based approach for promoting cardiometabolic health.

## Conclusion

To our knowledge, this is the first systematic review and meta-analysis to synthesize evidence from both cross-sectional and prospective observational studies on TRE and cardiometabolic health in adults. Current cross-sectional evidence suggests that restricting daily eating duration to ≤12 h was not consistently associated with cardiometabolic health. These findings complement robust evidence from RCTs and mechanistic insights from preclinical studies by highlighting the challenges of implementing TRE as a lifestyle factor in real-world populations. Together, the broader evidence base suggests that, while TRE has biological potential, its effectiveness at the population level remains to be established. Therefore, efforts to promote cardiometabolic health should continue to prioritize the well-established guidelines for preventing CMDs.

## Supplementary information

Supplementary Materials

## Data Availability

Data described in the manuscript will be made available from the first author (viktoria.feit@med.uni-tuebingen.de) upon reasonable request.
