# Supplementary material for: The cross-sectional and prospective associations between time-restricted eating and cardiometabolic health in community-dwelling adults: A systematic review and meta-analysis of observational studies
Source: Eur J Clin Nutr. 2026 May 23;80(8):747–62. doi: 10.1038/s41430-026-01755-w (PMC13493316; doi:10.1038/s41430-026-01755-w)
Supplement: Supplementary file 1 [file 41430_2026_1755_MOESM1_ESM.docx]

**Supplementary Materials**

**Supplementary Text 1**

**PubMed**

((("Fasting"[MeSH Terms] OR "Fasting"[Title/Abstract] OR "Time Restricted Eating"[Title/Abstract] OR "Time Restricted Feeding"[Title/Abstract] OR "intermittent energy restriction*"[Title/Abstract] OR "chrononutrition*"[Title/Abstract] OR "chrono nutrition*"[Title/Abstract] OR "eating duration*"[Title/Abstract] OR (("Circadian Clocks"[MeSH Terms] OR "circadian rhythm*"[Title/Abstract] OR "circadian clock*"[Title/Abstract]) AND ("eating"[Title/Abstract] OR "feeding"[Title/Abstract]))) AND ("Cardiometabolic Risk Factors"[MeSH Terms] OR "Cardiometabolic Risk*"[Title/Abstract] OR "cardiometabolic biomarker*"[Title/Abstract] OR "cardiometabolic effect*"[Title/Abstract] OR "cardiometabolic endpoint*"[Title/Abstract] OR "cardiometabolic outcome*"[Title/Abstract] OR "metabolic outcome*"[Title/Abstract] OR "Metabolic health"[Title/Abstract] OR ("Metabolic Syndrome"[MeSH Terms] AND "Cardiovascular Diseases"[MeSH Terms])) AND ("Epidemiologic Studies"[MeSH Terms] OR "case-control"[Title/Abstract] OR "case-control"[Title/Abstract] OR (("case"[Title/Abstract] OR "cases"[Title/Abstract]) AND ("control"[Title/Abstract] OR "controls"[Title/Abstract])) OR "cohort study"[Title/Abstract] OR "cohort analysis"[Title/Abstract] OR "follow-up study"[Title/Abstract] OR "follow-up study"[Title/Abstract] OR "observational study"[Title/Abstract] OR "longitudinal"[Title/Abstract] OR "retrospective"[Title/Abstract] OR "cross sectional"[Title/Abstract] OR "questionnaire"[Title/Abstract] OR "questionnaires"[Title/Abstract] OR "survey"[Title/Abstract] OR ("epidemiolog*"[Title/Abstract] AND ("study"[Title/Abstract] OR "studies"[Title/Abstract])))) NOT (("Pediatrics"[MeSH Terms] OR "Child"[MeSH Terms] OR "Infant"[MeSH Terms]) NOT "Adult"[MeSH Terms])) NOT ("Animals"[MeSH Terms] NOT "Humans"[MeSH Terms])

**Cochrane Library (CENTRAL)**

1	(exp Pediatrics/ or pediatric*.kw. or exp child/ or child*.kw. or exp infant/ or infant*.kw.) not (exp adult/ or adult*.kw.)	98721

2	exp animals/ not exp humans/	2963

3	exp fasting/ or fasting.ti,ab,kw. or time restricted eating.ti,ab,kw. or time restricted feeding.ti,ab,kw. or intermittent energy restriction*.ti,ab,kw. or chrononutrition*.ti,ab,kw. or chrono-nutrition*.ti,ab,kw. or (eating adj2 duration*).ti,ab,kw. or ((exp Circadian Clocks/ or circadian rhythm*.ti,ab,kw. or circadian clock*.ti,ab,kw.) adj5 (eating or Feeding).ti,ab,kw.)	41247

4	exp Cardiometabolic Risk Factors/ or (cardiometabolic adj2 risk*).ti,ab,kw. or cardiometabolic biomarker*.ti,ab,kw. or cardiometabolic effect*.ti,ab,kw. or cardiometabolic endpoint*.ti,ab,kw. or (cardiometabolic adj2 outcome*).ti,ab,kw. or metabolic Outcome*.ti,ab,kw. or metabolic health.ti,ab,kw. or (exp Metabolic Syndrome/ and exp Cardiovascular Diseases/)	4619

5	exp Epidemiologic Studies/ or case control.ti,ab,kw. or case-control.ti,ab,kw. or ((case or cases) and (control or controls)).ti,ab,kw. or cohort study.ti,ab,kw. or cohort analysis.ti,ab,kw. or follow up study.ti,ab,kw. or follow-up study.ti,ab,kw. or observational study.ti,ab,kw. or longitudinal.ti,ab,kw. or retrospective.ti,ab,kw. or cross sectional.ti,ab,kw. or questionnaire.ti,ab,kw. or questionnaires.ti,ab,kw. or survey.ti,ab,kw. or (epidemiolog* and (study or studies)).ti,ab,kw.	480400

6	3 and 4 and 5	358

7	6 not 1	325

8	7 not 2	325

**Web of Science (Core Collection)**

(TS=(adult$) AND TS=(fasting OR "intermittent fasting" OR ("time restricted" NEAR/0 (eating OR fasting)) OR "intermittent energy restriction$" OR chrononutrition$ OR chrono-nutrition$ OR (eating NEAR/2 duration) OR (("Circadian Clock$" OR "circadian rhythm$") NEAR/5 (eating OR feeding))) AND TS=((cardiometabolic NEAR/2 (risk$ OR outcome$)) OR (cardiometabolic NEAR/0 (biomarker$ OR effect* OR endpoint$)) OR "metabolic Outcome$" OR "metabolic health" OR ("metabolic syndrome$" AND "cardiovascular disease$")) AND TS=((epidemiologic NEAR/5 stud*) OR (case$ NEAR/5 control$) OR (cohort NEAR/0 (stud* OR analysis)) OR (("follow up" OR follow-up OR observational) NEAR/0 stud*) OR longitudinal OR retrospective OR "cross sectional" OR questionaire$ OR survey$)) NOT TS=(Animal$ NOT Human$)

**CINAHL**

((((MH "Fasting+") OR TI (fasting OR ("time restricted" N0 (eating OR feeding)) OR "intermittent energy restriction#" OR chrononutrition# OR chrono-nutrition# OR (eating N2 duration) OR (((MH "Circadian Rhythm+") OR "Circadian Clock#" OR "circadian rhythm#") N5 (eating OR feeding))) OR AB (fasting OR ("time restricted" N0 (eating OR feeding)) OR "intermittent energy restriction#" OR chrononutrition# OR chrono-nutrition# OR (eating N2 duration) OR (((MH "Circadian Rhythm+") OR "Circadian Clock#" OR "circadian rhythm#") N5 (eating OR feeding)))) AND ((MH "Cardiovascular Risk Factors+") OR TI ((cardiometabolic N2 (risk# OR outcome)) OR (cardiometabolic N0 (biomarker# OR effect* OR endpoint#)) OR "metabolic outcome#" OR "metabolic health" OR ((MH "Metabolic Syndrome X+") AND (MH "Cardiovascular Diseases+"))) OR AB ((cardiometabolic N2 (risk# OR outcome)) OR (cardiometabolic N0 (biomarker# OR effect* OR endpoint#)) OR "metabolic outcome#" OR "metabolic health" OR ((MH "Metabolic Syndrome X+") AND (MH "Cardiovascular Diseases+")))) AND ((MH "Epidemiological Research+") OR (MH "Cross Sectional Studies") OR TI ((epidemiologic N5 stud*) OR (case# N5 control#) OR (cohort N0 (stud* OR analysis)) OR (("follow up" OR follow-up OR observational) N0 stud$) OR longitudinal OR retrospective OR "cross sectional" OR questionnaire# OR survey#) OR AB ((epidemiologic N5 stud*) OR (case# N5 control#) OR (cohort N0 (stud* OR analysis)) OR (("follow up" OR follow-up OR observational) N0 stud$) OR longitudinal OR retrospective OR "cross sectional" OR questionnaire# OR survey#))) NOT (((MH "Child+") OR (MH "Adolescence+")) NOT (MH "Adult+"))) NOT ((MH "Animals+") NOT (MH "Human"))

**Supplementary Table 1:** Overview of adjustment made for analyses of cross-sectional studies and cross-sectional analyses of cohort studies evaluating the association between TRE and markers of cardiometabolic health. Confounders were collected from the maximally adjusted models that were reported in each of the studies.

| **Author (year)** | **age** | **education** | **sex** | **race/**  **ethnicity** | **smoking** | **BMI** | **physical activity** | **alcohol consumption** | **sleep duration** | **energy intake** | **other factors** |
| --- | --- | --- | --- | --- | --- | --- | --- | --- | --- | --- | --- |
| Ali et al. (2023) (48) | X | X | X | X | X | X |  | X | X | X | diabetes,  cohort |
| Bernardes da Cunha et al. (2023) (49) | X | X | X | X | X |  | X | X | X | X | household  income,  marital status |
| Currenti et al. (2021) (25) | X | X | X |  | X |  | X |  |  | X | occupational level, Mediterranean diet adherence score, breakfast, dinner |
| Estrada-deLeón et al.  (2022) (55) | X | X | X |  | X | X | X | X | X | X | sedentary behavior,  the MEDAS score, T2DM, hypertension, hyper-cholesterolemia |
| Ha and Song (2019) (50) | X | X |  |  | X | X^*^ | X | X |  | X | household income, type of work, survey period  ^*^(except for obesity and abdominal obesity) |
| Kwak et al. (2023) (17) | X | X |  |  | X | X | X | X |  |  | household income level, household type |
| Marinac et al. (2015) (44) and  Marinac et al. (2015)^1^ (45) | X | X |  | X |  |  |  |  |  |  |  |
| O’Connor et al. (2022) (51) | X | X | X | X |  |  |  |  | X |  | income, household size,  employment status, bedtime, ATUS survey cycle, season, day of week |
| Ueda et al. (2021) (29) | X |  | X |  | X |  | X^*^ | X | X^**^ | X | site, job grade,  marital status, overtime work,  sodium intake,  fruit intake,  vegetable intake, fat intake, carbohydrate intake,  ^*^(leisure-time and work related)  ^**^(weekday sleep duration) |
| Wirth et al. (2021) (26) | X |  | X | X | X | X |  | X | X | X | marital status, cholesterol medicine, days for dietary reports, DII |
| Zeng et al. (2023) (52) | X | X | X | X | X | X | X | X |  | X | marital status, ratio of family income:poverty,  diabetes,  HEI-2015 |
| Zeraattalab-Motlagh et al. (2022) (16) | X | X |  |  | X | X^*^ | X |  |  | X | marital status, occupation,  ^*^(except for abdominal obesity) |
| Makarem et al. (2020) (30) and  Makarem et al. (2021)^2^ (46) | X |  |  | X |  |  |  |  |  |  | health insurance |
| Alkhulaifi et al. (2024) (32) | X | X |  |  | X | X | X |  |  |  | income |
| Xiao et al. (2021) (54) | X |  | X | X |  |  | X^*^ |  | X^**^ | X | Chronotype, duration of sedentary time,  midpoint of overnight fasting,  % of TEI consumed within 2 h after wake up,  % of TEI consumed within 2 h before bedtime,  ^*^(total steps per day)  ^**^(total time in bed) |

^1^Marinac et al. (45) and Marinac et al. (44) were assessed as one study. ^2^Makarem et al. (30) and Makarem et al. (46) were evaluated as one study. Abbreviations: ATUS, American Time Use Survey; BMI, body mass index; DII, Dietary Inflammation Index; HEI-2015, Healthy Eating Index-2015; MEDAS, Mediterranean Diet Adherence Screener; TEI, total energy intake; T2DM, type 2 diabetes mellitus.

**Supplementary Table 2:** Overview of adjustment made for analyses of prospective and retrospective cohort studies evaluating the association between TRE and cardiometabolic health markers. Confounders were collected from the maximally adjusted models that were reported in each of the studies.

| **Author (year)** | **age** | **education** | **sex** | **race/**  **ethnicity** | **smoking** | **BMI** | **physical activity** | **alcohol consumption** | **sleep duration** | **energy intake** | **other factors** |
| --- | --- | --- | --- | --- | --- | --- | --- | --- | --- | --- | --- |
| Kahleova et al. (2017) (28) | X | X | X | X |  |  | X |  | X | X | marital status,  personal income,  dietary pattern,  television watching,  use of high blood pressure medications |
| Palomar-Cros et al. (2023) (27) and Palomar-Cros et al. (2023)^1^ (47) | X | X | X |  | X | X | X | X |  | X^*^ | family history of T2DM, healthy dietary pattern, Western dietary pattern, number of dietary records, number of eating, occasions,  time of first meal,  monthly income per unit of consumption, family history of CVDs, episodes of binge, drinking  ^*^(excluding alcohol) |
| Makarem et al. (2020) (30) and Makarem et al. (2021) (30)² | X |  |  | X |  |  |  |  |  |  | health insurance |
| Cheng et al. (2024) (53) | X | X | X | X | X | X | X^*^ | X | X | X | annual family income,  AHEI,  meal skipping,  whether dietary data was surveyed on weekend,  fasting plasma glucose, TG, TC,  drug use for controlling glucose/hypertension/ dyslipidemia,  family history of CVD  ^*^(regular exercise habits) |

^1^Palomar-Cros et al. (2023) (47) and Palomar-Cros et al. (2023) (27) were evaluated as one study. ^2^Makarem et al. (2020) (30) and Makarem et al. (2021) (46) were evaluated as one study. Abbreviations: AHEI, alternative healthy eating index; BMI, body mass index; CVDs, cardiovascular diseases; NR, not reported; TC, total cholesterol; TG, triglycerides; T2DM, type 2 diabetes mellitus.

n number of cross-sectional studies

| Quality assessment criterion | Yes | No | NR | NA |
| --- | --- | --- | --- | --- |
| Q1 | 12 | 0 | 0 | 0 |
| Q2 | 12 | 0 | 0 | 0 |
| Q3 | 5 | 2 | 2 | 3 |
| Q4 | 12 | 0 | 0 | 0 |
| Q5 | 0 | 10 | 1 | 1 |
| Q6 | 6 | 5 | 0 | 1 |
| Q7 | 2 | 10 | 0 | 0 |
| Q8 | 10 | 2 | 0 | 0 |
| Q9 | 10 | 2 | 0 | 0 |
| Q10 | 1 | 11 | 0 | 0 |
| Q11 | 12 | 0 | 0 | 0 |
| Q12 | 0 | 2 | 10 | 0 |
| Q13 | 0 | 0 | 0 | 12 |
| Q14 | 12 | 0 | 0 | 0 |

**Supplementary Figure 1:** Overview of the risk assessment of cross-sectional studies using the NIH quality assessment tool, presented as stacked bar charts. Quality assessment criteria: Q1, Research question; Q2 and Q3, Study population; Q4; Groups recruited from the same population and uniform eligibility criteria; Q5, Sample size justification; Q6, Exposure assessed prior to outcome measurement; Q7, Sufficient timeframe to see an effect; Q8, Different levels of the exposure of interest; Q9, Exposure measures and assessment; Q10, Repeated exposure assessment; Q11, Outcome measures; Q12, Blinding of outcome assessors; Q13, Follow-up rate; Q14, Statistical analyses.

n number of cohort studies

| Quality assessment criterion | Yes | No | NR | NA |
| --- | --- | --- | --- | --- |
| Q1 | 6 | 0 | 0 | 0 |
| Q2 | 6 | 0 | 0 | 0 |
| Q3 | 3 | 0 | 1 | 2 |
| Q4 | 6 | 0 | 0 | 0 |
| Q5 | 0 | 6 | 0 | 0 |
| Q6 | 5 | 1 | 0 | 0 |
| Q7 | 5 | 1 | 0 | 0 |
| Q8 | 6 | 0 | 0 | 0 |
| Q9 | 5 | 1 | 0 | 0 |
| Q10 | 3 | 3 | 0 | 0 |
| Q11 | 6 | 0 | 0 | 0 |
| Q12 | 0 | 1 | 5 | 0 |
| Q13 | 2 | 0 | 4 | 0 |
| Q14 | 6 | 0 | 0 | 0 |

**Supplementary Figure 2:** Overview of the risk assessment of cohort studies using the NIH quality assessment tool, presented as stacked bar charts. Quality assessment criteria: Q1, Research question; Q2 and Q3, Study population; Q4; Groups recruited from the same population and uniform eligibility criteria; Q5, Sample size justification; Q6, Exposure assessed prior to outcome measurement; Q7, Sufficient timeframe to see an effect; Q8, Different levels of the exposure of interest; Q9, Exposure measures and assessment; Q10, Repeated exposure assessment; Q11, Outcome measures; Q12, Blinding of outcome assessors; Q13, Follow-up rate; Q14, Statistical analyses.
